# Overview of Nrf2 as Therapeutic Target in Epilepsy

**DOI:** 10.3390/ijms160818348

**Published:** 2015-08-07

**Authors:** Liliana Carmona-Aparicio, Claudia Pérez-Cruz, Cecilia Zavala-Tecuapetla, Leticia Granados-Rojas, Liliana Rivera-Espinosa, Hortencia Montesinos-Correa, Jacqueline Hernández-Damián, José Pedraza-Chaverri, Aristides III Sampieri, Elvia Coballase-Urrutia, Noemí Cárdenas-Rodríguez

**Affiliations:** 1 Laboratory of Neurochemistry (Neurosciences), National Institute of Pediatrics, D.F. 04530, Mexico; E-Mail: lgranados_2000@yahoo.com.mx; 2 Laboratory of Neuroplasticity and Neurodegeneration, Cinvestav, D.F. 07360, Mexico; E-Mail: cau9@yahoo.com; 3 Laboratory of Physiology of the Reticular Formation, National Institute of Neurology and Neurosurgery-MVS, D.F. 14269, Mexico; E-Mail: cztecua@yahoo.com.mx; 4 Laboratory of Pharmacology, National Institute of Pediatrics, D.F. 04530, Mexico; E-Mail: lili_rives@yahoo.com; 5 Service of Endocrinology, National Institute of Pediatrics, D.F. 04530, Mexico; E-Mail: hortenciamontesinoscorrea@yahoo.com; 6 Department of Biology, Faculty of Chemistry, National Autonomous University of Mexico, D.F. 04150, Mexico; E-Mails: jdamian@email.ifc.unam.mx (J.H.-D.); pedraza@unam.mx (J.P.-C.); 7 Department of Comparative Biology, Faculty of Sciences, National Autonomous University of Mexico, D.F. 04150, Mexico; E-Mail: aris_sampieri@yahoo.com.mx

**Keywords:** Nrf2, stress oxidative, transcription factor nuclear, epilepsy

## Abstract

Oxidative stress is a biochemical state of imbalance in the production of reactive oxygen and nitrogen species and antioxidant defenses. It is involved in the physiopathology of degenerative and chronic neuronal disorders, such as epilepsy. Experimental evidence in humans and animals support the involvement of oxidative stress before and after seizures. In the past few years, research has increasingly focused on the molecular pathways of this process, such as that involving transcription factor nuclear factor E2-related factor 2 (Nrf2), which plays a central role in the regulation of antioxidant response elements (ARE) and modulates cellular redox status. The aim of this review is to present experimental evidence on the role of Nrf2 in this neurological disorder and to further determine the therapeutic impact of Nrf2 in epilepsy.

## 1. Overview of Oxidative Stress and Nrf2

Oxidative stress is a state of imbalance in the production of reactive oxygen (ROS) and nitrogen (RNS) species and the antioxidant defenses. Since the 1970s, oxidative stress has been associated with diverse physiological and pathological states; ROS and RNS can be both beneficial and harmful in biological systems [1,2,3].

In particular, microglial cell activation and impaired mitochondrial function are common pathological characteristics of many diseases and contribute to the increased generation of ROS. Oxidative damage and mitochondrial dysfunction are currently accepted as key hallmarks of classic inflammatory, degenerative, systemic, and neuronal diseases [4,5]. ROS are small biological molecules that are incompletely reduced or have oxygen with different electronic distributions and are more reactive than molecular oxygen. ROS perform diverse physiological functions and play important roles in host defense, cell signaling, gene expression regulation, and cell differentiation. ROS levels are tightly regulated in the central nervous system (CNS) because the brain is particularly sensitive to oxidative stress [5,6,7]. When CNS ROS levels increase as a result of pathological conditions, the cellular antioxidant capacity is overwhelmed and results in oxidative stress and damage to essential cellular structures, as observed in Parkinson’s disease [8,9,10].

To counteract the detrimental effects of ROS and to restore the delicate redox balance in the CNS, cells are equipped with endogenous antioxidant defense mechanisms that comprise several antioxidant enzymes. The production of many antioxidant enzymes is regulated at the transcriptional level by the transcription factor nuclear factor (erythroid-derived 2)-like 2 (Nrf2) [11,12]. Accumulating evidence in experimental models of different disorders suggests that Nrf2 pathway activation represents a promising therapeutic approach to restore the systemic and neuronal redox balance by reducing ROS-mediated neuronal damage. However, only a few Nrf2-activating compounds have been tested in a clinical setting. To maintain the physiological redox balance, cells are endowed with a selection of endogenous antioxidant enzymes. The transcription of these cytoprotective proteins is controlled by Nrf2, which plays a central role in the regulation of cellular redox status [11,12,13].

Under homeostatic conditions, Nrf2 transcription is repressed by its negative regulator, Kelch-like ECH-associated protein 1 (Keap1) [13]. Following exposure to ROS, Nrf2 dissociates from cytosolic Keap1 and translocates to the nucleus. It subsequently binds to antioxidant response elements (ARE) in the promoter region of hundreds of genes that are involved in antioxidant protection and detoxification, including superoxide dismutase (SOD) [14], glutathione peroxidases [15], peroxiredoxins [16], heme oxygenase-1 (HO-1) [17,18], and NAD(P)H:quinone oxidoreductase-1 (NQO1) [19,20,21,22]. Together, these free radical scavenging enzymes create a powerful antioxidant defense mechanism. Currently, various natural and synthetic Nrf2-activating compounds have been identified, and the number of reports that describe the beneficial effects of Nrf2 activation in experimental models of neurodegeneration has steadily increased. This therapeutic option is based on the activation of Nrf2-driven antioxidant gene expression in the CNS, which thereby limits oxidative stress and oxidative cellular injury and prevents disease progression.

### 1.1. Structural Characteristics of Nrf2 Protein

Nrf2 is a cap‘n’collar (CNC) basic-region leucine zipper (bZIP) transcription factor that modulates the cellular redox status. Nrf2 was identified in 1994 as a cDNA clone encoding a transcriptional activator that binds to the tandem NF-E2/AP1 repeat of the β-globin locus control region [23]. The protein Nrf2 is ubiquitously expressed in all human and mouse tissues, although at different levels in different tissues. Nrf2 binds to DNA as a heterodimer with the small musculoaponeurotic fibrosarcoma (Maf) proteins, MafF, MafG and MafK [24,25,26]. Together, Maf and Nrf2 are able to recognize ARE-DNA sequences to induce transcription of phase II antioxidant enzymes. ARE-regulated genes include HO-1, NQO1 [27] and glutathione *S*-transferases, as well as glutathione-synthesizing enzymes, glutamate-cysteine ligase catalytic subunit (GCLC) and glutamate-cysteine ligase modulatory subunit (GCLM) [28,29]. Currently, Nrf2 is recognized to regulate more than 200 genes, including those involved with mechanisms of cytoprotection, intermediary metabolism, and mitochondrial function.

Antioxidants work as strong activators of Nrf2. After metabolizing, they produce a small amount of oxidative stress that triggers Nrf2 activation. For this reason, they are typically used to study the mechanisms involved in Nrf2 activation. However, Nrf2 activation is complex and requires several phases to mediate cytoprotective gene expression as a mechanism of antioxidant defense [30,31,32,33]. In addition to the antioxidant response via the modulation of the expression of numerous detoxifying and antioxidant genes, Nrf2 regulates genes that control various processes, such as immune and inflammatory responses, carcinogenesis and metastasis, cognitive dysfunction, and addictive behavior [34,35].

**Figure 1:**
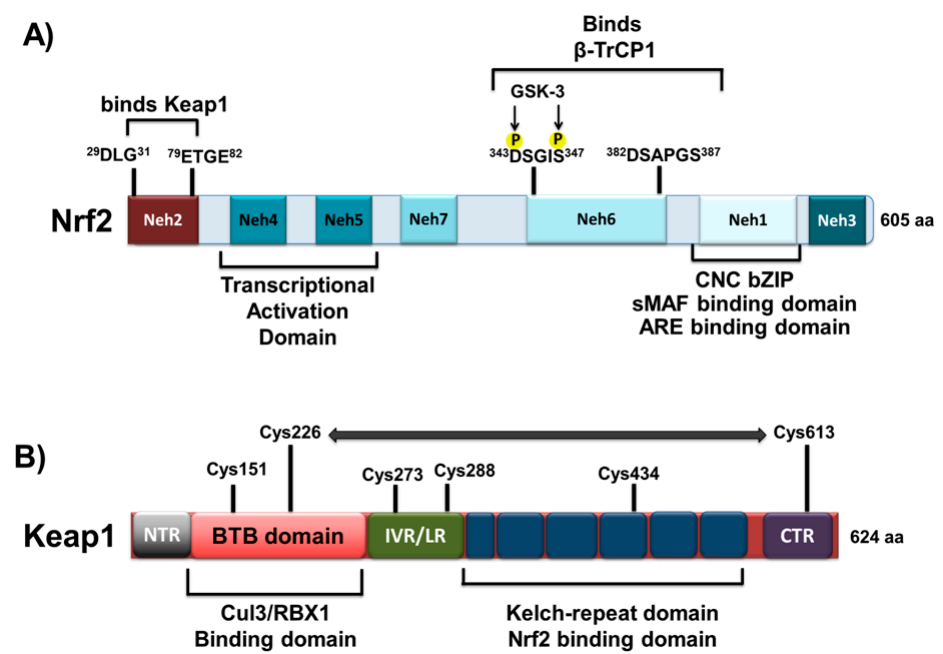
Domain structures of the transcription factor Nrf2 and its repressor Keap1. **(A**) The proposed positions of the Nrf2-ECH homology 1–7 domains are indicated. The DLG and ETGE motifs in the Neh2 domain control the interaction with Keap1; the number of amino acids involved is indicated. The Neh6 domain binds to β-TrCP1 adaptor protein; the DSGIS motif is phosphorylated by GSK-3β prior to binding with β-TrCP1, but not DSAPGS motif. The Neh1 CNC-bZIP domain is responsible for dimerization with small musculoaponeurotic fibrosarcoma (Maf) proteins and is required for binding to ARE sequences in DNA; (**B**) Domains of the Keap1 protein. The BTB domain is indicated as a red box, which is required for the formation of Keap1 homodimers as well as the recruitment of Cullin-3 (Cul3) or RBX1. The Kelch-repeat domain, indicated as a blue box, controls Nrf2 interaction. The C-terminal region (CTR) is depicted in purple. The region between the BTB and Kelch repeat domains constitute the intervening region (IVR). Amino acid residues that function as a sensor for electrophiles and H_2_O_2_ (Cys-151, Cys-226/Cys-613, Cys-273/Cys-288 and Cys-434) are shown. The horizontal two-headed arrow, between Cys-226 and Cys-613, means that they form a disulfide bridge when exposed to H_2_O_2_. *Abbreviations*: BTB, bric-a-brac, tramtrack, broad-complex domain; bZip, basic region leucine zipper; CNC, cap‘n’collar; NTR, N-terminal region; CTR, carboxyl terminal region; Cullin 3, Cul3; DGR, double glycine repeat; IVR, intervening region; GSK-3β glycogen synthase kinase-3β; Keap1, Kelch-like ECH (erythroid cell-derived protein with CNC homology)-associated protein 1; LR, linker region; Maf, musculoaponeurotic fibrosarcoma protein; Neh, Nrf2-ECH homology; Nrf2, nuclear factor erythroid-2 related factor 2; RBX1, ring-box 1 E3 ubiquitin protein ligase.

The protein structure of Nrf2 is composed of seven domains, Nrf2-ECH homology (Neh) domains 1–7 (Figure 1). The CNC-bZIP region is localized inside the Neh1 domain and after dimerization with small Maf proteins, binds to DNA [27,35]. The Neh2 domain interacts with Keap1 [36]. The resultant Keap1-Nrf2 complex is a cellular sensor for oxidative stress that mediates the transcriptional activation of Nrf2.

Keap1 is a substrate adaptor component of a Cul3-dependent E3 ubiquitin ligase complex that induces Nrf2 downregulation through proteasomes. The Keap1 protein comprises 624 amino acids and contains two important domains that forms the Bric-a-brac, tramtrack, broad-complex/poxivirus zinc finger (BTB/POZ), which has been described in protein dimerization. Keap1 forms a dimer with Nrf2 at an Nrf2:Keap1 stoichiometric ratio of 1:2. This dimer recognizes and binds two degrons, DLG and ETGE in Nrf2, to mediate the repression of Nrf2 activity [37]. The intervening region/linker region (IVR/LR) is located between these two important domains. Along the BTB/POZ and IVR/LR domains are localized reactive cysteine residues. Under stressed conditions, Keap1 binds Nrf2 and promotes rapid Nrf2 downregulation through the ubiquitin proteasome pathway. However, after exposure to oxidative and electrophilic stresses, reactive cysteine residues are modified. These processes prevent the protein degradation of Nrf2 and promote its transcriptional activity by repressing Keap1 [38]. Recent findings have described alternative Nrf2 protein degradation mechanisms independent of Keap1. The Neh6 domain of Nrf2 exhibits DSGIS and DSAPGS motifs that recruit the dimeric β-transducin repeat containing protein (β-TrCP), a substrate adaptor for the S-phase kinase-associated protein 1 (Skp1)-Cul1-Rbx1 core E3 complex [39]. Moreover, the DSGIS motif is phosphorylated by glycogen synthase kinase (GSK-3β), necessary for interactions with β-TrCP to induce the downregulation of Nrf2 protein [40].

In a basal state, the Nrf2/Keap1/Cul3/RBX1 complex remains in the cytosol and continuously degrades Nrf2 in a Keap1 independent manner. Nrf2 protein degradation could be mediated by the phosphorylation of GSK-3β and β-TrCP. Moreover, the Nrf2 protein is also present in the nucleus, in complex with Keap1/Cul3/RBX1. However, the stability of Nrf2 is modulated by other intracellular mediators such as Fyn, Src, Yes, and Fgr. These kinases phosphorylate Nrf2 at Tyr568, inducing the nuclear export and degradation of Nrf2 [31,32,33].

The involvement of Nrf2 has been confirmed using genetically-modified mice that lack the obtained factor Nrf2^-/-^. The Nrf2^-/-^ mice exhibited an apparently normal development; thus, it was concluded that Nrf2 was essential for murine erythropoiesis, growth, and development. However, these mice were unable to induce the expression of genes responsible for carcinogen detoxification and protection against oxidative stress, particularly the phase II genes NQO1 (a flavoprotein), glutamate cysteine ligase (GCL), glutathione *S*-transferase (GST), and HO-1 [27,36,41,42,43]. More recent studies have demonstrated that Nrf2 also contributes to 26S proteasome activity, which is critically involved in NRF1 and Nrf2 protection against oxidative stress and xenobiotic responses [41].

### 1.2. Nrf2 and Oxidative Stress: Signal Transduction and Gene Regulation

Nrf2 constitutes the main oxidative stress response in cells. Under basal conditions, Nrf2 binds to the Keap1 homodimer and is ubiquitinated in the cytosol by the adaptor protein for the Cullin 3 (Cul3)-dependent E3 ubiquitin ligase, where is subsequently degraded [44]. The Keap1 protein dimer senses cellular oxidative stress by releasing Nrf2 to the nucleus. In the nucleus, Nrf2 forms a complex with Maf and Jun proteins and binds to the ARE sequences [28,45,46] in the upstream promoter region present in many antioxidant genes; with this binding, Nrf2 initiates gene transcription. Following recovery of cellular redox homeostasis, Keap1 translocates to the nucleus to dissociate Nrf2 from ARE, which results in Nrf2 degradation. Thus, Nrf2 acts as a primary cellular defender against the cytotoxic effects of oxidative stress. Furthermore, the seven functional domains (Neh1–Neh7) of Nrf2 have various roles. Neh1 contains a CNC-type bZIP DNA-binding motif that allows Nrf2 to bind DNA and dimerize with other transcription factors [23].

Neh2 is a major regulatory domain and is located in the N terminus of Nrf2. Neh2 contains seven lysine residues responsible for ubiquitin conjugation [47], as well as two binding sites, the ETGE and DLG motifs. These binding sites regulate Nrf2 stability and interact with Keap1 [48].

The Neh3, Neh4, and Neh5 domains interact with coactivators to enable the transactivation of Nrf2 target genes. The Neh3 domain binds to the chromo-ATPase/helicase DNA-binding protein family member CHD6, which functions as an Nrf2 transcriptional coactivator [49]. The Neh4 and Neh5 domains have been demonstrated to interact with the CH3 domains of CREB binding protein to facilitate the transactivation of Nrf2 target genes [50].

The Neh6 domain contains two binding sites for the β-transducin repeat-containing protein (β-TrCP), which are referred to as DSGIS and DSAPGS motifs. β-TrCP acts as a substrate adaptor for the Skp1–Cul1–Rbx1/Roc1 ubiquitin ligase complex. The phosphorylation of the DSGIS motif by GSK-3 increases the ability of β-TrCP to ubiquitinate Nrf2 and promotes its rapid turnover [51,52].

Two mechanistic models have been proposed for Nrf2 regulation: The hinge and latch model and the Keap1-Cul3 dissociation model [48,53]. In the hinge and latch model, the two binding motifs of the Neh2 domain in Nrf2 have different affinities [48]. Keap1 proteins must occupy both the DLG and ETGF motifs to lead to ubiquitination. Modifications of the cysteine residues of Keap1 by ROS lead to the release of the DLG motif (latch) without changes in the ETGF motif (hinge) on Neh2. The release of the DLG motif inhibits ubiquitin ligase. The Keap1-Cul3 dissociation model is based on the disruption of the interaction between the Keap1-Cul3 E3 ligase without changes in the conformation of Keap1 [53].

Evidence further suggest that the Nrf2-Keap1 system contributes to protection against various diseases, such as cancer, liver toxicity and inflammation [41], and has relevance in the physiopathology of various neurological diseases, such as Alzheimer’s disease [54], Parkinson’s disease [34], multiple sclerosis [55], amyotrophic lateral sclerosis [56], Huntington’s disease [35], and epilepsy [57].

## 2. Epilepsy and Nrf2

### 2.1. Generalities of Epilepsy

Epilepsy is a complex, chronic neurological disorder characterized by increased and abnormal synchronization of neuronal electrical activity, which is manifested as seizures [58]. Seizure refers to a behavioral change (impaired motor and/or sensory activity) as a result of synchronicities, which are alterations in the rhythmic firing of a neuronal population [59,60]. The International League Against Epilepsy (ILAE) has defined Epilepsy as “a condition characterized by two or more recurrent epileptic seizures over a period longer than 24 h, unprovoked by any immediate identified cause” [61,62].

Epidemiological studies indicate that this pathology affects approximately 1% of the world [63,64]. The incidence is greater at early ages, higher in childhood and in the final decades of life, and stabilizes in adulthood; temporal lobe epilepsy is the most common manifestation of these neurological diseases (40%) [65,66,67]. Currently, up to 30% of temporal lobe epilepsy patients have seizures despite treatment with antiepileptic drugs, either mono- or polytherapy, which result in physical harm to the patient and psychosocial dysfunction. This results in physiological damage in the forms of electrolyte imbalance, cerebral edema, congestive renal failure, refractory status epilepticus (SE), and sudden death [68] and also restricts their quality of life and performance in society [69,70,71,72]. Therefore, it is a priority to continue investigating the mechanisms underlying epilepsy to identify new targets and potential therapeutic agents for the treatment of this disorder.

### 2.2. Mechanisms Involved in Epilepsy

Epilepsy is a heterogeneous syndrome in which all cells of the nervous system, including glia [73], are involved. Various types of seizures are triggered by diverse alterations: Electrophysiological (paroxysmal depolarization changes); morphologic (anatomical, histological or ultrastructural); neurochemical (neurotransmitters and receptors); ionic (impaired sodium and potassium concentrations, the activity of the sodium/potassium pump or other ion currents); and metabolic and endocrine alterations [74,75].

Currently, it is believed that the initiation and propagation of paroxysmal discharges involve: The ability of a group of neurons to generate discharges; an excitatory glutamatergic system capacity, especially by the activation of *N*-methyl-d-aspartate (NMDA), which amplifies the signal and facilitates the generation of excitatory postsynaptic potentials (EPSPs); and the inhibitory GABAergic system capacity to regulate NMDA receptor activation to prevent the genesis of intracerebral transmission and control its spread via the generation of inhibitory postsynaptic potentials (IPSPs) [74,75,76,77].

**Figure 2:**
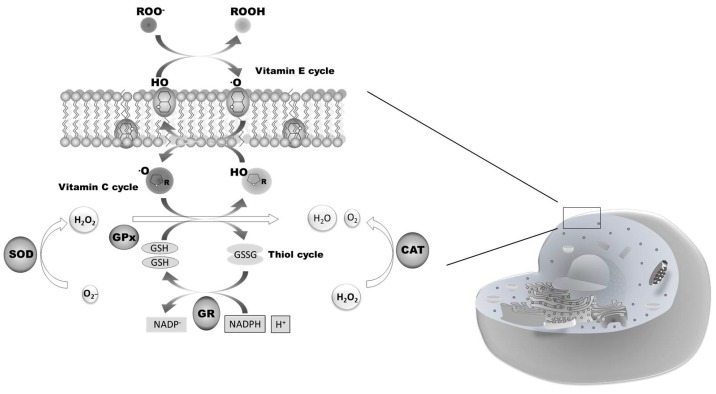
Biochemical pathways involved in the antioxidant response. Reactive Oxygen Species (ROS) production is accompanied by the activation of the enzymes involves in ROS scavenging, such as superoxide dismutase (SOD), catalase (CAT), ascorbate-glutathione cycle enzimes (GR, GPx), and gluthatione reductase (GR). The antioxidant enzymes show different patterns of activity considering the ROS production.

Sustained neuronal electrical activity and seizures can lead to neuronal injury and death resulting from underlying biochemical mechanisms such as the formation of excessive ROS (Figure 2) [78]. This leads to oxidative stress-induced abnormal structural alterations of cellular proteins, membrane lipids, DNA, and RNA.

Oxidative stress is an underlying mechanism in the initiation and progression of epilepsy and also contributes to neuronal degeneration in the epileptic focus [79,80]. Antioxidants have been suggested as therapeutic strategies for the treatment and modulation of epilepsy.

In the 1990s, oxidative stress was initially associated with neuronal hyperexcitability in neurological diseases [81,82].

The first experimental evidence of the involvement of oxidative stress in epilepsy was reported by Armstead’s group [83]. In their experiment, cerebral superoxide anion generation during bicuculline-induced seizures was measured in newborn pigs (1–6 days, 0.9–2.1 kg). Using two closed cranial windows inserted over the parietal cortices, SOD-inhibitable nitroblue tetrazolium (NBT) reduction was determined as an index of superoxide anion generation in separate groups of piglets under different conditions: 20 min without prior administration of bicuculline; the first 20 min of bicuculline-induced (5 mg/kg, *i.v.*) seizure generation in piglets treated with vehicle (saline); and the first 20 min of bicuculline-induced seizures in piglets pretreated with indomethacin trihydrate (5 mg/kg *i.v*.). SOD-inhibitable NBT reduction was increased in the piglets subjected to bicuculline-induced seizure activity (2.4 ± 0.6 pmoL/mm^2^ in 20 min) compared with the control piglets (0.4 ± 0.3 pmoL/mm^2^ in 20 min). Pretreatment with indomethacin (5 mg/kg *i.v*.) reduced the SOD-inhibitable NBT reduction during seizures to the control level (0.5 ± 0.4 pmoL/mm^2^ in 20 min). The authors concluded that the newborn pig brain produces small quantities of superoxide anion radicals during bicuculline-induced seizures; furthermore, cyclooxygenase metabolism of arachidonic acid appears to be a major source of these radicals [83].

Subsequent studies supported the involvement of oxidative stress in epilepsy, particularly in SE induced by kainic acid [84,85] and pilocarpine [85]. Dalton’s group reported that severe brain damage caused by SE induced by kainic acid was, in part, caused by oxidative stress [84]. The authors demonstrated that kainic acid induced an increase in metallothionein-I and HO-1 mRNA, as well as increases in c-fos, heat shock protein-70, and interleukin-1β mRNA, with little or no change in metallothionein-III, Mn-SOD, Cu-Zn-SOD, glutathione-*S*-transferase or GPx mRNA levels. The induction of metallothionein-I and HO-1 gene expression suggests that oxidative stress is induced by kainic acid-induced seizures. Furthermore, the induction of interleukin-1β gene expression suggests an inflammatory response in brain regions damaged by kainic acid-induced seizures [84].

Dal-Pizzol’s group was the first to study lipid peroxidation levels through determination of the levels of thiobarbituric acid reactive substances (TBARS), early and late after SE, which were induced by pilocarpine or kainic acid. The authors determined that in the hippocampus, there was a slight (12.04% and 14.32%) enhancement in the TBARS levels measured 12–14 h after the end of SE induced by pilocarpine and kainic acid, respectively. The mean TBARS levels in the pilocarpine-treated animals were significantly decreased (27.29% and 25.33%, respectively, 7–9 and 75–80 days after SE induced by pilocarpine). In the same period after kainic acid-induced SE, the TBARS levels were not different compared with the control group. These results suggest the involvement of ROS in kainic acid and pilocarpine-induced SE [85].

Other studies also support the role of oxidative stress in epileptogenesis [86,87] and in seizure-induced cell damage [86,87,88,89,90].

Frantseva’s group used amygdala-kindled rats to examine the generation of ROS following seizures and their contributions to seizure development and seizure-induced neuronal loss. They measured the free radical levels (malonaldehyde and 4-hydroxy-2(*E*)-nonenal) and found that lipid peroxidation increased in both hemispheres of kindled rats *vs*. sham-operated controls. Furthermore, the authors observed increased cell death in all hippocampal areas. They also demonstrated that the administration of antioxidants (vitamin E and glutathione) prevented increased lipid peroxide levels and hippocampal neuronal death during electrical kindling and did not have an effect on seizure development. The authors concluded that epileptiform activity resulted in free radical production and could be considered as a factor that induces cell death [86].

In 2014, a meta-analysis study by Martinc’s group confirmed an association between epilepsy and the increase of lipid peroxidation. They proposed that during the epileptic process, neuroprotective treatments with antioxidants could lead to less severe structural damages, reduced epileptogenesis and decreased cognitive deterioration [87].

However, Liang’s group indicated a role of mitochondrial superoxide radical-mediated oxidative damage in seizure-induced neuronal death. They found that a systemic administration of kainate (s.c.) in rats increased mitochondrial superoxide production resulting in neuronal death and increased 8-hydroxy-2-deoxyguanosine (an oxidized lesion of DNA) levels in the hippocampus of treated rats. Also, they found that a catalytic antioxidant (manganese (III) tetrakis(4-benzoic acid)porphyrin) inhibited kainate-induced mitochondrial superoxide production, 8-hydroxy-2-deoxyguanosine formation and neuronal loss in the rat hippocampus. The authors demonstrated a role for mitochondrial superoxide production in hippocampal pathology induced by kainate seizures. Furthermore, Liang’s group suggested that mitochondrial superoxide radical-mediated oxidative stress plays a relevant role in excitotoxicity induced by kainate seizures and that the antioxidants may be used in the therapeutic treatment of seizure disorders [88]. In 2002, Kovacs’s group confirmed that repeated seizure-like event (SLEs) in hippocampal slice cultures (experimental model of status epilepticus in slice) decreased the intracellular and intra-mitochondrial Ca^2+^ signals (despite unaltered Ca^2+^ influx) and also decreased mitochondrial depolarization and the NAD(P)H signal. They also found that the free radical scavenger, α-tocopherol, protected the slice cultures against this damage and reduced the ongoing impairment of NAD(P)H production. They finally suggested the involvement of ROS of mitochondrial origin in epileptic cell damage and that free radical scavenging may prevent epilepticus–induced cell loss [89].

In summary, in experimental models of epilepsy, studies on increased ROS production and oxidative damage to cellular targets continue. Mitochondrial dysfunction, apoptotic factors, and ROS production and detoxification have been demonstrated in human and experimental models. However, no definitive conclusion can yet be made on the occurrence of mitochondrial dysfunction following seizures and/or epileptogenesis [90].

The study of oxidative stress in epilepsy has been based on experimental models [83,84,85,91,92,93,94,95] and in epileptic patients [92,93,94,95,96,97,98,99,100,101].

Currently, these studies are focused on the modulation of oxidative stress, particularly through the Nrf2-ARE signal pathway.

### 2.3. Nrf2 in Epilepsy

Recent studies demonstrated that the Nrf2-ARE signaling pathway could represent an important target in protecting the brain from the damage induced by ischemic stroke [102,103] and kainate toxicity [57]. However, the protection afforded by activating Nrf2-ARE signal pathway has not been fully studied in epilepsy. There are only a few studies that have evaluated this pathway as a therapeutic target in epileptic seizures.

The Nrf2 pathway has been considered for the treatment of epilepsy due to the presence of oxidative stress from altered steady-state glutathione levels during epileptogenesis [90]. Moreover, the ketogenic diet profoundly alters redox processes, in part by increasing cellular glutathione levels via Nrf2 pathway activation [104]. Therefore, a clear rationale exists for validating the Nrf2 pathway as antiepileptic or antiepileptogenic therapies.

Wang and coworkers [105] evaluated whether Nrf2-ARE signaling pathway activation could protect the brain from seizure-mediated damage and ameliorate cognitive impairment and oxidative stresses induced by epileptic seizures. Wistar rats and Nrf2-deficient or control mice underwent chronic amygdala kindling by electrical stimulation. Sulforaphane (SF) was used to activate the Nrf2-ARE signaling pathway [105]. Pretreatment with SF markedly decreased the after discharge duration (ADD) compared with the kindling group. SF ameliorated the cognitive impairment induced by epileptic seizures when the rats were tested using the Morris water maze. Additionally, SF led to a noticeable decrease in the concentration of malondialdehyde (MDA) and a significant increase in the glutathione levels compared with the kindling group [91]. Western blot analyses of the Nrf2 protein levels in the nucleus and the HO-1 and NQO1 protein levels in the cytoplasm extracted from the hippocampus and real-time fluorescence quantitative polymerase chain reaction (PCR) mRNA expression analyses indicated that the pretreatment with SF led to significant increases in both the protein and mRNA levels of Nrf2, HO-1 and NQO1 compared with the control and kindling groups. Nrf2 deficiency (Nrf2-knockout mice) significantly worsened the epileptic seizures and cognitive impairment caused by amygdala kindling and also obliterated the protective effect of SF, which was observed in the control mice (Nrf2^+/+^ mice). These results demonstrated that Nrf2-ARE signaling pathway activation could increase the expression of antioxidant enzymes (HO-1 and NQO1) and suppress the progression of amygdala kindling. Furthermore, the activation ameliorated the cognitive impairment and oxidative stresses induced by epileptic seizures. Therefore, the activation of the Nrf2-ARE signaling pathway may be one strategic target for epilepsy therapies [105].

Wang and coworkers [106] also observed the activation mechanisms of the Nrf2-ARE signaling pathway and two Nrf2-regulated gene products (HO-1 and NQO1) after seizures. In their study, Wistar rats were rapidly kindled in the amygdala via electrical stimulation (temporal lobe epilepsy model). Twenty-four hours after the last seizure, the hippocampi of the control, sham and kindled rats were examined for oxidative stress parameters (MDA and GSH) and the expressions of Nrf2, HO-1, and NQO1 at the protein and gene levels. Seizures induced an increase in MDA levels and a decrease in GSH levels in the hippocampus of the kindled rats [106]. The protein levels of Nrf2 in the nucleus and HO-1 in the cytoplasm were significantly increased, and the mRNA levels of Nrf2 and two Nrf2-regulated gene products (HO-1 and NQO1) were also up-regulated in the hippocampus after seizure. It could be concluded that seizures induced oxidative stresses in the hippocampi of kindled rats, activating the Nrf2-ARE signaling pathway and resulting in the up-regulation of antioxidant and detoxifying enzymes, which may reduce oxidative damage [106]. Using transcription factor enrichment analysis (TFEA) on publically available gene expression data sets (using IPA software) from a variety of epilepsy-related studies (including animal epilepsy models and ketogenic diet-treated animals), Mazzuferi and coworkers [107] identified Nrf2 as one of the main transcription factors with a potential therapeutic use. Human hippocampal tissue obtained from treatment-resistant patients with temporal lobe epilepsy (TLE) who underwent temporal lobe resection exhibited increased Nrf2 mRNA levels, which was consistent with the bioinformatic analysis. Because of the limited availability and heterogeneity of human tissue, Nrf2 expression and its downstream genes were also studied in a mouse model of TLE, in which mice develop spontaneous recurrent seizures (SRS) following the induction of SE with an injection of pilocarpine. There was a strong induction of Nrf2 and its target genes (*i.e.*, HO-1, and NQO1) in the early stages following SE induction [107]. To examine the role of Nrf2 activation in epilepsy, Nrf2 overexpression was induced via an injection of adeno-associated virus that coded for human Nrf2 in the hippocampi of mice with SRS. The findings indicated a reduction in the number and duration of generalized seizures and increased neuronal survival and the normalization of astroglial activation [107].

**Figure 3:**
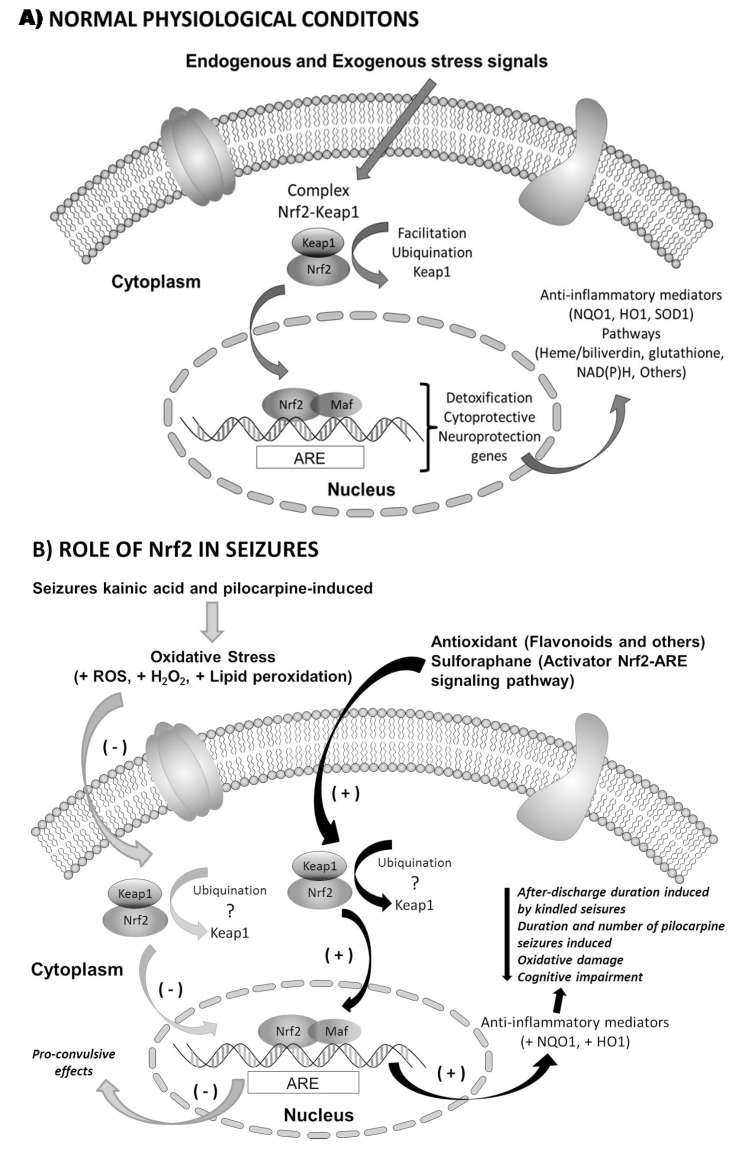
Role of Nrf2 in basal conditions to response as endogenous and exogenous stimulus (**A**) and in epilepsy state as consequences of kainic acid and pilocarpine-induced seizures as examined in human hippocampal tissue from resistant patients with temporal lobe epilepsy (**B**). Note that stabilization mechanisms have not yet been identified. In addition, antioxidants promote increased Nrf2 mRNA levels in the cell.

Li and coworkers [108] demonstrated that glutamate-induced oxidative damage is a major contributor to pathological cell death within the nervous system. This research group identifying phytochemicals that have intrinsic antioxidant effects against glutamate-induced oxidative stress, demonstrated that lindenenyl acetate (isolated from the roots of *Lindera strychnifolia*) increased the cellular resistance of HT22 cells to oxidative injury caused by glutamate. This effect occurred through Nrf2/ARE-dependent HO-1 expression via extracellular signal-regulated kinase (ERK) pathway activation. The authors investigated whether the lindenenyl acetate treatment of HT22 cells induced the translocation of Nrf2 to the nucleus. Using Western blot analyses, they examined the presence of Nrf2 proteins in the nuclear fractions of HT22 cells. Additionally, HT22 cells transiently transfected with the ARE-luciferase plasmid were exposed to lindenenyl acetate, and changes in luciferase activity were used as a measure of ARE activation [108]. The reporter assays indicated that lindenenyl acetate dose-dependently increased ARE-driven luciferase activity, and ARE activation was strongly correlated with the increase in HO-1 activity [109]. Because several studies had reported that MAPK pathway activation contributes to the induction of HO-1 [96], Li and coworkers examined the effect of lindenenyl acetate on MAPK activation in HT22 cells. Cells were exposed to lindenenyl acetate, the total protein was harvested, and Western blots were subsequently performed using anti-phospho-c-Jun NH2-terminal kinase (JNK), ERK1/2 and p38 antibodies. At a concentration of 40 μM, which strongly induced HO-1 levels, lindenenyl acetate activated the ERK pathway and increased ERK phosphorylation [108].

To investigate the role of MAPK in HO-1 expression and to test the cytoprotective effects of lindenenyl acetate, Li and coworkers examined the effects of specific inhibitors of ERK1/2 (U0126), JNK (SP600125), and p38 (SB203580) on the HO-1 levels using Western blots and MTT assays. The ERK MAPK pathway inhibitor significantly reduced lindenenyl acetate-induced HO-1 expression, whereas the JNK and p38 inhibitors did not reduce the expression. As expected, the ERK MAPK pathway inhibitor abolished lindenenyl acetate-induced cytoprotection, whereas the inhibitors of the JNK or p38 MAPK pathways did not exhibit this change. These results suggest that lindenenyl acetate increases the cellular resistance to glutamate-induced oxidative injury in mouse hippocampal HT22 cells, presumably through the ERK pathway-Nrf2/ARE-dependent HO-1 expression. HO-1 expression by pharmacological modulators may represent a useful target for therapeutic intervention because HO-1 has been proposed to play an important cellular defense role against oxidant injury [108]. Kraft and coworkers [57] was the first group that examined the contributions of the Nrf2-ARE pathway in limiting kainate-induced neurodegeneration using mice that expressed a human placental alkaline phosphatase (hPAP) reporter construct in response to ARE activation (ARE–hPAP mice) and mice with disrupted Nrf2 (Nrf2 KO mice). ARE induction following KA-induced neurodegeneration was observed; the Nrf2 KO animals exhibited enhanced behavioral symptoms and increased mortality in response to kainate-induced seizure activity (temporal lobe epilepsy model), as well as pyramidal neuron loss and cellular infiltration caused by kainite [57]. This was the first observation of an increased sensitivity of mice lacking Nrf2 to kainate. Furthermore, the relevance of Nrf2 in this model of epilepsy creates a tenable linkage to nearly all neurodegenerative diseases because the vast majority of these pathologies involve an excitotoxicity component. The modification of select aspects of the Nrf2-ARE system may serve to limit seizure severity and aid the intrinsic response of the brain to neuronal damage [57]. These results validate the overall Nrf2 pathway as a target for treatment of temporal lobe epilepsy and other related epilepsies. The development of Nrf2 activators and its downstream gene products could be considered as promising therapeutic targets. We have summarized the experimental evidence of the role of Nrf2 in epilepsy in Figure 3.

## 3. Therapeutic Relevance

The experimental evidence suggests that Nrf2 could be a promising therapeutic target for chronic neurological diseases like epilepsy [110]. Exploiting this natural defense mechanism could lead to an innovative therapeutic approach for different types of epilepsy, especially temporal lobe epilepsy, and where it is demonstrated that Nrf2 has a relevant role, e.g., in alternative therapies like ketogenic diets [111]. It is also necessary to continue to investigate the role of Nrf2 in this neurological disease, in epileptogenesis and seizure-induced cell death and to determine whether its effects on oxidative stress are a cause or consequence of seizures. Finally, studies will be necessary to determine the relevance of Nrf2 in neuro-inflammation processes and its role in intractable epilepsy.

Antioxidant and detoxification genes are associated with Nrf2 and its ability to help neuronal cells cope with toxic insults and oxidative stress. Various antioxidant agents could provide neuroprotective effects in a variety of neurological diseases through the activation of the Nrf2-ARE pathway. Recent findings have shown the therapeutic efficacy of natural and synthetic Nrf2 inducers in the treatment of epilepsy and other neurodegenerative diseases. These therapeutic strategies could be used in the management and treatment of associated degenerative changes and cognitive alterations.
